# The Future of Dentistry

**Published:** 1896-09

**Authors:** F. F. Fletcher

**Affiliations:** St. Louis.


ARTICLE VIII.

### THE FUTURE OF DENTISTRY.

BY DR. F. F. FLETCHER, ST. LOUIS.

I dreamed. I thought it morning and that I must arise at once and vulcanize a case before breakfast.

I was thinking this and other portions of the day's work over, mentally arranging the details and incidentally indulging my laziness, when suddenly there came a knocking at my chamber door, and a voice in no uncertain tone informed me I had overslept, and to arise at once as a gentleman wished to see me. Hastily I made my toilet and entered my drawing-room to receive my caller. His card showed him to be a brother dentist.

He informed me that, in answer to my advertisement, he came to apply for the position of assistant. Being pleased with his appearance I informed him I would try him, and he might go to work at once. Introducing him to the laboratory, I remarked that he might vulcanize that case while I went to breakfast, he having eaten earlier. Imagine my surprise to see his eyes open widely and hear him remark excitedly:

"Is that a vulcanizer?"

Thinking I had a practical joker to deal with I turned on my heel saying: "I'll be back in about an hour."

"Hold on there." said he, "I am going to;" and before I could say a word he had gotten out of that laboratory and slammed the door shut, looking about as nervous as a junior student about to extract his first tooth. "Oh! I have heard of those steam bombshells that used to blow up and kill people when making plates was a part of every dentist's work, and not wishing to endanger my life or return to the practices of fifty years ago, think I will try to find a position in a modorn office where I can practice what I have been taught."

Thinking I had a harmless lunatic to deal with I could not resist the temptation to quiz him a little. So I remarked :

"Doctor, before you go will you kindly tell me what you mean by modern dentistry, where you received your education, etc ?"

"Why certainly," said he, "I am an American by birth, born, raised and educated in Havana, State of Cuba, U. S. A., and graduated A. D. 1950."

Now I was sure he was crazy, but I was becoming interested, and said to him :

"And you never saw a vulcanizer ?"

"No, and never expect to except in a museum or mechanical office ?"

"What do you make plates out of, out there ?"

"Why we don't make any except a few for country people and for foreigners. In nearly every large city there is still to be found a mechanical dentist and such work is referred to him."

"Have the teeth quit decaying out there ?"

"No, doctor; the teeth do not decay nearly so much as they did formerly, but likely would if we permitted them; but by our system fully 90 per cent. of the people of twenty-five years of age have never lost a tooth. People having sound teeth or even filled teeth, the mouth being kept clean and free from caries, will have less trouble with their children's teeth. You know the child may inherit good tooth structure, imperfectly fused fissures, etc., but must be inoculated with caries, usually transmitted by kissing. You know the adage 'the more kissing the more caries,' and America leads the world in both. So you see with 90 per cent. of the teeth in good shape modern dentistry is strictly operative, a pleasure to the operator and a blessing to humanity. Plates are rare, bridge-work a curiosity, crowns scarce, toothache and abscesses occur but seldom, facial neuralgia nearly exterminated, root filling —that skeleton in every old dentist's closet—buried, and whose specter walks but seldom when by accident a pulp dies and the root must be filled. The dentist is no longer a bugbear to school children, and the ruthless destruction of the first permanent molar has ceased forever. This is not local but done all over the United States, and all brought about by our system in the short space of fifty years. Truly the advances of dentistry in the past fifty years stand without a parallel in the history of the world."

He was waxing eloquent over his pet theme, so I asked him to outline the system which brought this all about.

"With pleasure. Under the old regime dentists knew their inability to save the teeth of the masses lay in their failure to secure the school children and even those under school age. How was it to be done? Some thought cheap dentistry, well advertised, would reach the masses. Accordingly they inserted columns, portraying in word pictures, their phenomenal successes, painless operations and prices within the reach of all. Many came to them and in most cases the quality of a filling diminished in proportion as the size of the cavity increased. Good teeth were sacrificed for poor crowns or barbarously extracted for still poorer plates.

"Whichever method promised the richest fee was adopted. In time the victims discovered that they had been duped and immediately decried dentistry in all forms. So this method was a signal failure at saving teeth. Few going to an office till driven by pain. How were they to be reached before this fatal warning and in time to save pulp and tooth?

Some enthusiasts tried public lectures; societies distributed pamphlets on how to care for children's teeth. Others tried articles in the daily papers to educate the masses. Various schemes were tried; and while all did some good the large majority of children under twelve years of age had never been in a dental office.

"The problem was, How are these children to be reached? About this time various large cities passed a law requiring every child to pass a physical examination on admission to school. The weak points were noted and the teachers of physical culture did what they could to correct them. A great change for the better was soon perceived. Another law was soon added, *viz:* a careful examination of the eyes by competent oculists. Many were the defects discovered, and thousands of eyes were saved that would otherwise have been ruined or permanently injured before their school days ended.

"Educators, scientists and professional men soon saw that a grand step had been taken for humanity. Dentists were not long in taking their cue, and by the energetic work of societies a plan was adopted and presented to the solons. Soon the edict went forth that the teeth must also be examined on the pupil's admission to school and at the beginning of every school year, unless they present a certificate from the dentist showing the teeth to be properly cared for. The only thing compulsory was the examination. The dentist examined the teeth, and his assistant made notes on a printed chart calling attention to each one's needs. These charts were given to parents by pupils and returned to teachers with signature of parent or guardian attached. Generally the parents would be astonished, and say, "Why I had no idea there was anything to do with my child's tooth or I would have had it attended to." The result you can easily imagine. Children came in such numbers that dentists were overrun. There were not enough to do half the work. Colleges boomed and had to double their capacities. In a short time few children feared the dentist because their teeth were not permitted to reach the painful point. For the first two or three years the examination took some time, but the dentists gave it gratuitously and were amply repaid for it. Soon the children began bringing in their certificates, signed by their dentist, which exempted them from examination and the work was reduced in proportion. The key-note had been struck; not by preaching to parents and children that the teeth should be attended to, but by opening each one's mouth and pointing out the various defects; for every child who was spoken to about it would declare the teeth were all right, unless they had had toothache at some time, and even then would generally deny it through fear of being hurt worse by the dentist. In 50 per cent. of those examined the children were shown and made to understand for the first time in their lives that they needed a dentist's services, and 90 per cent. of the parents were as ignorant of the children's needs as they themselves. But when they found out positively that such services were needed, it was surprising how soon the young hopefuls began coming into the dental offices. The mother generally accompanied the child on the first visit, and told the dentist about the chart being sent from school, expressed her surprise that anything was wrong with darling's teeth, and requested him to make an examination in order to verify or refute the official report. It always ended by the dentist receiving a new patient, or the promise of one, and instructions to do all necessary work. Then the battle was won. After getting their teeth put in shape, cleaned, etc., it was but little trouble for the dentist to arouse their interest and pride. It became a fad among the children; they pointed with pride to their clean teeth and little fillings. These little patients became the dentist's best friends, for they kept reminding the laggards, and soon those who deferred the visit and whose teeth showed inattention were driven to the office by public opinion of school-mates. Often younger brothers and sisters came with the older children and wanted their teeth 'fixed.' They were cared for of course, but in case nothing was needed they frequently showed displeasure at not being permitted to have a tooth filled. 'But, doctor, I must be going and allow you to breakfast.' I thanked him for his kindness and invited him to call again and inform me more as to detail, which he promised to do. As the door closed rather loudly I heard another and well-known voice inquire if I knew it was 7.30 o'clock. I awoke from slumber and to the fact that I really must arise, vulcanize the case myselt and resume my practice, not along the beautiful lines I had seen in dreamland but according to the old methods in use in 1895."—*Dental Review.*

